# How to enhance primary healthcare capacity? Evidence from Chinese provinces through a configurational lens

**DOI:** 10.3389/fpubh.2025.1706057

**Published:** 2025-11-19

**Authors:** Fang Wang, Yi Wang, Jiayi Yang, Liqing Li

**Affiliations:** 1 School of Economics Management and Law, Jiangxi Science and Technology Normal University, Nanchang, China; 2 Research Center for Health Policy and Development, Jiangxi Science and Technology Normal University, Nanchang, China; 3 International Business School, Hainan University, Haikou, China

**Keywords:** primary healthcare capacity, multidimensional collaborative governance framework, driving pathways, configurational analysis, fsQCA

## Abstract

**Objectives:**

This study aims to investigate the conditional configurations and influencing mechanisms that contribute to the enhancement of primary healthcare service capacity by examining the effects of seven factors: economic development level, urbanization rate, population size, health expenditure, medical insurance fund revenue, education level, and the implementation of tiered medical care systems.

**Methods:**

Guided by systems theory, resource dependence theory, and institutional logic theory, this study develops a multidimensional collaborative governance analytical framework. Using fuzzy-set Qualitative Comparative Analysis (fsQCA) with 31 provincial-level regions in mainland China as cases, it investigates the configurational pathways and interactive effects of seven conditions—economic development level, urbanization rate, population size, healthcare expenditure, medical insurance fund revenue, educational attainment, and hierarchical diagnosis and treatment (HDT)—on PHC capacity.

**Results:**

The analysis reveals two key findings. First, none of the individual conditions constitutes a necessary prerequisite for strong PHC capacity. However, high healthcare expenditure consistently emerges as a core condition across all four configurations, underscoring that sustained financial investment is a central component in multiple pathways for strengthening PHC capacity. Second, three distinct configurational patterns are identified as drivers of PHC capacity improvement in China: (1) the “Resource-Intensive Pathway,” characterized by a resource compensation effect; (2) the “Fiscal-Led Pathway,” representing a single-core driving pattern; and (3) the “Institutional-Synergy Pathway,” which combines resource endowment with institutional arrangements. Third, the pathways leading to weak PHC capacity are not mere inverses of the successful ones. The analysis identified two independent failure modes: “Systemic Resource Scarcity,” characterized by the fatal dual absence of healthcare expenditure and medical insurance funds, and “Institutional-Resource Double Failure,” where the lack of fiscal investment coincides with failed HDT reforms.

**Conclusion:**

The study recommends that local governments in China reinforce fiscal guarantees and adopt context-specific, adaptive pathways to achieve systemic improvements and balanced development in PHC capacity. The findings offer important theoretical and practical insights into the driving pathways for enhancing PHC capacity in China.

## 1 Introduction

Primary healthcare (PHC) capacity forms a core foundation for safeguarding public health and serves as a critical pillar in building a high-quality, efficient healthcare resource system. In 2023, the General Offices of the Central Committee of the Communist Party of China and the State Council issued the *Opinions on Further Deepening Reform to Promote the Healthy Development of the Rural Healthcare System*, explicitly emphasizing that strengthening the rural healthcare system is an urgent requirement for advancing the Healthy China initiative. In August 2025, the National Development and Reform Commission, in collaboration with the National Health Commission and other relevant departments, launched the Medical and Healthcare Strengthening Project to address deficiencies in PHC services. At present, China’s PHC system faces multiple challenges, including imbalanced resource distribution and suboptimal service efficiency (1, 2). In particular, amid rapid urbanization and economic restructuring, disparities in PHC capacity across regions have become increasingly pronounced (3, 4). Against this backdrop, identifying the factors influencing PHC capacity and leveraging multidimensional collaborative governance models to enhance it has become a pressing issue of shared concern for both scholars and policymakers.

As a core component of the national public health system, PHC capacity has been examined from diverse perspectives. This reflects evolving demands in health governance. Numerous studies have highlighted that factors such as economic development level, urbanization rate, population size, healthcare expenditure, medical insurance fund revenue, educational attainment, and the implementation of HDT significantly influence PHC capacity (3, 5–8). As these factors do not operate in isolation, their interdependencies can form various configurational combinations that jointly shape PHC capacity. Adopting a configurational perspective enables a deeper understanding of the complex mechanisms that underlie PHC capacity enhancement across provinces. This study takes the 31 provincial-level regions of mainland China (excluding Hong Kong, Macao, and Taiwan) as its cases. Drawing on the practical context of provincial efforts to promote PHC capacity, it develops a multidimensional collaborative governance framework grounded in systems theory, resource dependence theory, and institutional logic theory. Employing fuzzy-set qualitative comparative analysis (fsQCA), the study examines the effects of seven factors—economic development level, urbanization rate, population size, healthcare expenditure, health insurance fund revenue, educational attainment, and HDT—on PHC capacity, aiming to identify the configurational conditions and underlying mechanisms that drive its improvement.

The theoretical contributions of this study are threefold. As the first point, it constructs a multidimensional collaborative governance framework, advancing theoretical integration in research on PHC capacity. In practice, the enhancement of PHC capacity is fundamentally driven by multidimensional interactions among resources, institutional arrangements, and the broader social environment. By integrating systems theory, resource dependence theory, and institutional logic theory, this study proposes a framework that systematically elucidates the interactive dynamics among seven categories of conditions influencing PHC capacity. We contend that these three theories are complementary rather than mutually exclusive. They operate at different analytical levels to provide a unified explanation: systems theory outlines the macro-structural context, resource dependence theory elucidates the core resource channels, and institutional logic theory reveals the micro-institutional and behavioral mechanisms. This integrated framework allows us to move beyond siloed explanations and capture the configurational nature of PHC capacity. The second point is that the study deepens our understanding of the mechanisms underlying the pathways to enhanced PHC capacity, revealing which configurations of conditions can promote improvement through the principle of “equifinality,” and further identifying the most critical conditions for PHC capacity enhancement. This approach overcomes the traditional paradigm of analyzing isolated conditions and demonstrates the dynamic adaptability and flexibility of pathways for improving PHC capacity. Lastly, it extends the application of the qualitative comparative analysis (QCA) method and offers methodological innovation in healthcare policy research. Current studies on healthcare services predominantly rely on multivariate regression, spatial econometric models, and structural equation modeling (7, 9, 10). These methods exhibit clear limitations in revealing complex causal relationships and interactions among variables, often yielding findings that describe surface-level phenomena without sufficiently uncovering deeper mechanisms. By employing fuzzy-set qualitative comparative analysis (fsQCA) to PHC capacity research, this study identifies four equivalent driving pathways across cases and systematically analyzes the causal asymmetry among conditions (11, 12).

## 2 Literature review and research framework

### 2.1 Literature review

PHC, as a fundamental pillar of national healthcare system, has received significant attention from numerous governments. As early as the 1960s, China established a primary healthcare network based on the commune system, centered around “barefoot doctors,” cooperative medical schemes, and brigade health clinics, which covered over 90% of the rural population nationwide. In academic circles, both domestic and international scholars have primarily analyzed PHC capacity from three perspectives: resource allocation, technological empowerment, and equity.

#### 2.1.1 Resource allocation

The allocation of primary healthcare resources in China has increased substantially over the years; however, pronounced inter-provincial disparities in resource distribution persist (3, 4), and the efficiency of medical and health services exhibits a degree of spatial correlation across cities (13). In some resource-rich regions, allocation efficiency is comparatively low, whereas in resource-scarce areas, efficiency tends to be higher (14). Resource allocation models and geographical distance significantly influence PHC capacity. This leads to marked disparities between urban peripheries and city centers. Therefore, urban planning strategies are needed to mitigate such gaps (15). Healthcare service efficiency is shaped by both the external regional environment and the internal operational context of hospitals, with specific determinants including inpatient volume, outpatient pharmaceutical expenditure, regional population density, demographic aging, the number of registered nurses, and fiscal autonomy (16). Educational attainment significantly influences healthcare service efficiency (5). Multiple pathways exist for the efficient allocation of health resources, with healthcare facilities playing a universal role, and allocation efficiency exhibiting causal asymmetry (10). To promote equitable and orderly resource circulation, governments should establish a well-calibrated system of incentives and penalties (17).

#### 2.1.2 Technology empowerment

Digital technologies are profoundly transforming the supply model of PHC capacity, propelling the healthcare system toward high-quality development by optimizing production factors, improving service quality, and enhancing governance mechanisms (18–20). From an organizational resilience perspective, digital technologies enhance the capacity to sense environmental changes, facilitate the effective absorption and integration of knowledge, and promote the systemic, coordinated allocation of resources. This process not only improves the scientific rigor of managerial decision-making but also optimizes the resource allocation structure of primary healthcare institutions, while enhancing the flexibility and sustainability of their organizational management models (21). Digital technologies can transform healthcare by integrating resources and fostering cross-regional collaboration. They disrupt traditional system monopolies, reshape care-seeking patterns, and enable more precise, efficient, and intelligent service delivery (22). The development of the digital economy exerts a significant positive impact on PHC capacity, with more pronounced effects in underdeveloped regions (23). The digitalization of healthcare services can continuously drive the equalization of PHC services (24). However, reliance solely on healthcare digitalization yields only limited improvements in PHC capacity (21). The process of digital transformation is far from smooth and faces multiple challenges, including data security concerns, technological adaptation barriers, impediments to doctor-patient communication, regulatory balancing dilemmas, and the need to optimize payment processes (24). In response to these challenges, Sarac proposed a series of countermeasures: strengthening data security and privacy protection, enhancing technological learning and adaptability, balancing technological application with clinical expertise, improving data governance and operational workflows, increasing patients’ digital literacy, reconciling standardization with innovation, and optimizing payment processes (23).

#### 2.1.3 Equity

Research on the equity of healthcare resources centers on the universal value of PHC. The allocation of scarce medical resources to ensure equal access to high-quality healthcare for all citizens constitutes a critical goal of socio-economic development. This issue encompasses multiple dimensions, including the distribution of medical resources, financial burdens, and the needs of vulnerable populations (25). Inequalities in healthcare service systems persist between urban and rural areas, primarily due to the disproportionate allocation of medical and health resources (4). In rural areas, population-dense regions experience supply–demand mismatches, while remote areas face substantially lower accessibility due to geographical barriers (26). Regionally, the greatest disparities in healthcare resource availability occur between cities in eastern China, whereas internal disparities are smallest in the western regions (4). Agricultural populations and older adults face systemic disadvantages in accessing healthcare resources, resulting in pronounced inequities (27, 28).

In summary, the aforementioned studies have made valuable contributions to enriching and deepening the understanding of PHC capacity and have advanced research in this field. However, these studies do not adequately account for the complex interdependencies and interactions among factors, nor how these interrelationships collectively enhance PHC capacity. In the context of China’s healthcare system, the conditions under which these influencing factors operate remain to be systematically examined and clearly delineated. Moreover, there is a pressing need for research offering stronger explanatory power to guide practice and promote the sustained enhancement of PHC capacity.

### 2.2 Research framework

**Figure 1:**
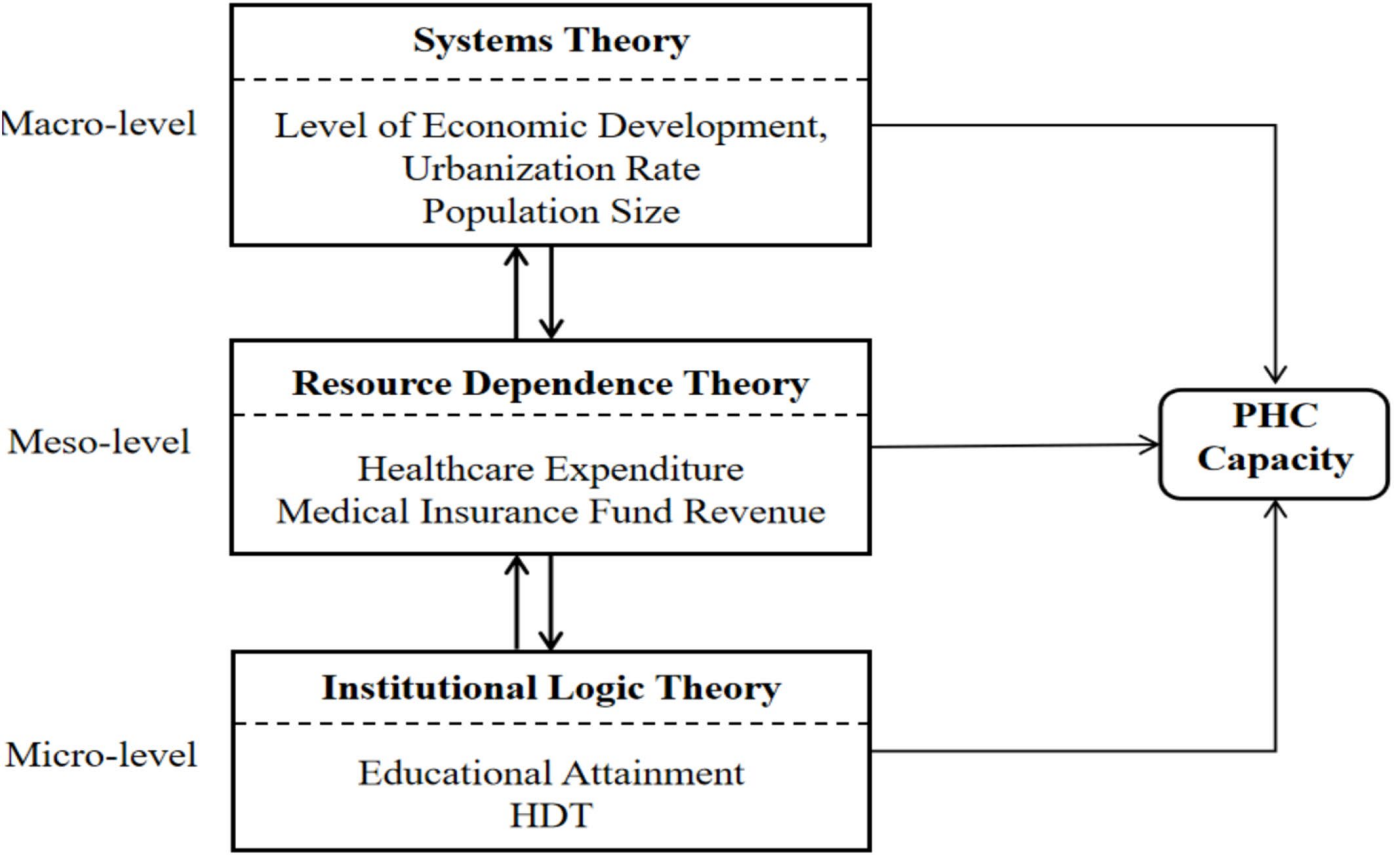
Theoretical analytical framework for PHC.

Although both domestic and international scholars have increasingly examined issues related to PHC capacity (9, 15, 29, 30), exemplary models or reference cases that could serve as practical benchmarks for enhancing PHC capacity remain scarce. Existing research reveals three primary limitations. To begin with, there is insufficient theoretical integration. Some studies adopt a purely resource-dependence perspective, emphasizing the decisive role of resource endowments while overlooking other determinants (6, 16), whereas others focus primarily on institutional logic theory, underscoring institutional constraints (21). Such approaches do not adequately account for the complex interactions and synergistic mechanisms among economic foundations, social capital, and institutional environments, thereby limiting the theoretical guidance available for identifying effective pathways to strengthen PHC capacity. Additionally, there is an underestimation of the interdependence among conditions, as much of the existing literature assumes symmetric relationships between independent and dependent variables, thereby constraining the identification of effective configurational pathways for improving PHC capacity. Third, PHC capacity depends on specific combinations of multiple conditions and on the logical relationships between these conditions and their outcomes (31). Specifically, it examines which combinations of conditions lead to improved service capacity (the emergence of the outcome) and which combinations result in diminished service capacity (the disappearance of the outcome). Notably, the conditions leading to the absence of high PHC capacity are not simply the mirror opposites of those leading to its presence. The configurations that enhance PHC capacity often differ from those that weaken it (12, 32). To address these limitations, this study develops a multidimensional collaborative governance framework grounded in systems theory, resource dependence theory, and institutional logic theory. In this study, we do not treat the three theories as parallel or isolated. Instead, we interweave them within a multilevel analytical logic. This integrated approach jointly explains the formation and enhancement of PHC capacity. Systems theory operates at the macro level, providing the structural context and environmental boundaries—defined by economic development, urbanization, and demographic pressure—within which primary healthcare institutions function. Resource dependence theory works at the meso level, elucidating how organizations obtain and manage critical resources (such as fiscal healthcare expenditure and insurance fund revenues) to ensure operational stability and service delivery within that systemic environment. Institutional logic theory functions at the micro level, uncovering how different logics—professional, governmental, and market-oriented—shape organizational behavior, policy implementation, and the utilization of resources, exemplified by the education level and hierarchical diagnosis and treatment (HDT) system. This integrated perspective allows us to analyze how factors from different domains—the macro-environment, resource inputs, and institutional arrangements—interact synergistically or substitutively to shape PHC capacity. The following sections elaborate on the role of each theory, while Figure 1 visually represents their interconnections within the unified model.

#### 2.2.1 Systems theory (macro-level)

Systems theory posits that organizational effectiveness arises from the nonlinear interactions between internal and external system components. This theory defines the structural context and environmental boundaries within which primary healthcare institutions operate. The macro-level conditions—Level of Economic Development (resource supply foundation), Urbanization Rate (socio-structural characteristic), and Population Size (service demand pressure)—set the stage but do not directly determine capacity; they enable or constrain the effectiveness of resources and institutions. Economic development provides the foundational resource base for PHC capacity. In economically advanced regions, higher local fiscal revenues enable more robust investment in infrastructure, medical equipment procurement, and personnel remuneration within PHC institutions, thereby fostering a “high investment–high capacity” feedback loop (3). Rapid urbanization and population growth in large cities substantially increase the demand for healthcare services. While urban cores tend to exhibit satisfactory healthcare resource availability and service quality, rapidly urbanizing peripheral areas often experience a pronounced mismatch between resource availability and service quality (14, 33). Although accelerated urbanization may enhance the efficiency of healthcare resource concentration, excessive population concentration can exacerbate the supply–demand imbalance (27).

#### 2.2.2 Resource dependence theory (meso-level)

Resource dependence theory posits that organizational sustainability is contingent upon its ability to acquire external resources. This theory explains how primary healthcare institutions acquire and manage critical external resources to ensure operational stability within the systemic environment. The meso-level conditions—Healthcare Expenditure (fiscal capacity) and Medical Insurance Fund Revenue (payment system support)—represent the essential lifeblood for PHC institutions, directly fueling service delivery. Government healthcare expenditure has been shown to exert a significant positive effect on the medical resources (34). Increased public healthcare spending enables primary healthcare institutions to procure advanced medical equipment, maintain adequate pharmaceutical reserves, and recruit sufficient numbers of highly qualified medical personnel, thereby improving disease diagnosis and treatment capabilities and enhancing the overall provision of healthcare services. Expanding government healthcare expenditure also helps reduce inequalities in access to healthcare resources in underdeveloped regions (4, 35). Reforms in health insurance payment mechanisms—such as the adoption of global budget payment systems—have effectively enhanced the financial stability of PHC institutions, enabling them to allocate healthcare resources more flexibly and ultimately strengthen their capacity (36). Healthcare expenditure and health insurance fund revenue operate synergistically through a “fiscal-social” dual-channel mechanism, thereby reinforcing the sustainability of PHC services.

#### 2.2.3 Institutional logic theory (micro-level)

The institutional logic uncovers how competing and complementary institutional logics shape organizational behavior, policy implementation, and the utilization of resources. The micro-level conditions—Educational Attainment (human capital logic influencing workforce supply and quality) and Hierarchical Diagnosis and Treatment (HDT) (policy intervention logic governing service delivery patterns)—determine how effectively systemic contexts and resources are translated into actual service capacity. Specifically, the impact of education levels on the supply of healthcare human resources reflects the “human capital logic.” As a key indicator of human capital accumulation, educational attainment (talent capital reserve) directly influences the supply of primary healthcare personnel. The educational background and continuing education opportunities available to primary healthcare providers determine their diagnostic capabilities and service quality (37). HDT embodyies the interaction between the policy intervention logic (government-led) and the market logic (patient choice). As an institutional innovation for optimizing healthcare resource allocation efficiency, HDT reflects the “government governance logic.” In China, the HDT is grounded in “health needs” and aims to achieve “collaborative governance,” operating along two practical logic lines: the health needs logic and the collaborative governance logic (38). HDT shift service provision from hospitals to community health service institutions—where major illnesses are treated in hospitals while minor conditions are addressed at the community level—thereby enhancing the overall PHC capacity (13, 39, 40).

Collectively, these theories offer complementary explanatory power: systems theory answers the “where and when” question by delineating structural context; resource dependence theory addresses “what” resources drive system performance; and institutional logic theory explicates “how and who” operationalize these resources within institutional settings. Together, they constitute an integrated multidimensional collaborative governance framework that underpins the configurational analysis in this study. This unified perspective enables the identification of cross-level synergies and compensatory mechanisms through which diverse condition configurations lead to enhanced PHC capacity.

## 3 Research methods and data

### 3.1 Qualitative comparative analysis (QCA)

Given the structural characteristics of primary healthcare resource allocation systems, fsQCA offers notable methodological advantages. For one thing, at the theoretical modeling level, fsQCA integrates analyses of necessary conditions and combinations of sufficient conditions, enabling the effective identification of nonlinear interactions among multiple factors and the detection of equifinal causal pathways leading to specific PHC capacity configurations (41). For another, in terms of data applicability, unlike traditional quantitative methods that often require large-scale datasets, fsQCA is particularly suitable for in-depth analysis of small-to-medium-sized samples (41, 42). Most importantly, its configurational logic transcends the assumption of linear relationships among variables; through Boolean algebra algorithms, it identifies the synergistic effects of key factors, thereby systematically unpacking the complex causal mechanisms arising from multidimensional interactions. The fsQCA method is particularly suited to explain the occurrence of a specific outcome, even when that outcome is operationalized through a key proxy variable (43). The focus on causal configurations allows for a nuanced understanding of how different conditions combine to lead to high (or low) levels of this specific, consequential aspect of PHC performance.

It is important to note that the primary objective of this fsQCA is to identify the configurational pathways that are sufficient for a high level of PHC capacity across different provinces at a specific point in time. This approach is inherently focused on uncovering causal complexity across cases rather than temporal dynamics within them. Therefore, a carefully constructed cross-sectional dataset is a well-established and appropriate means to address our research question (43).

**Figure 2:**
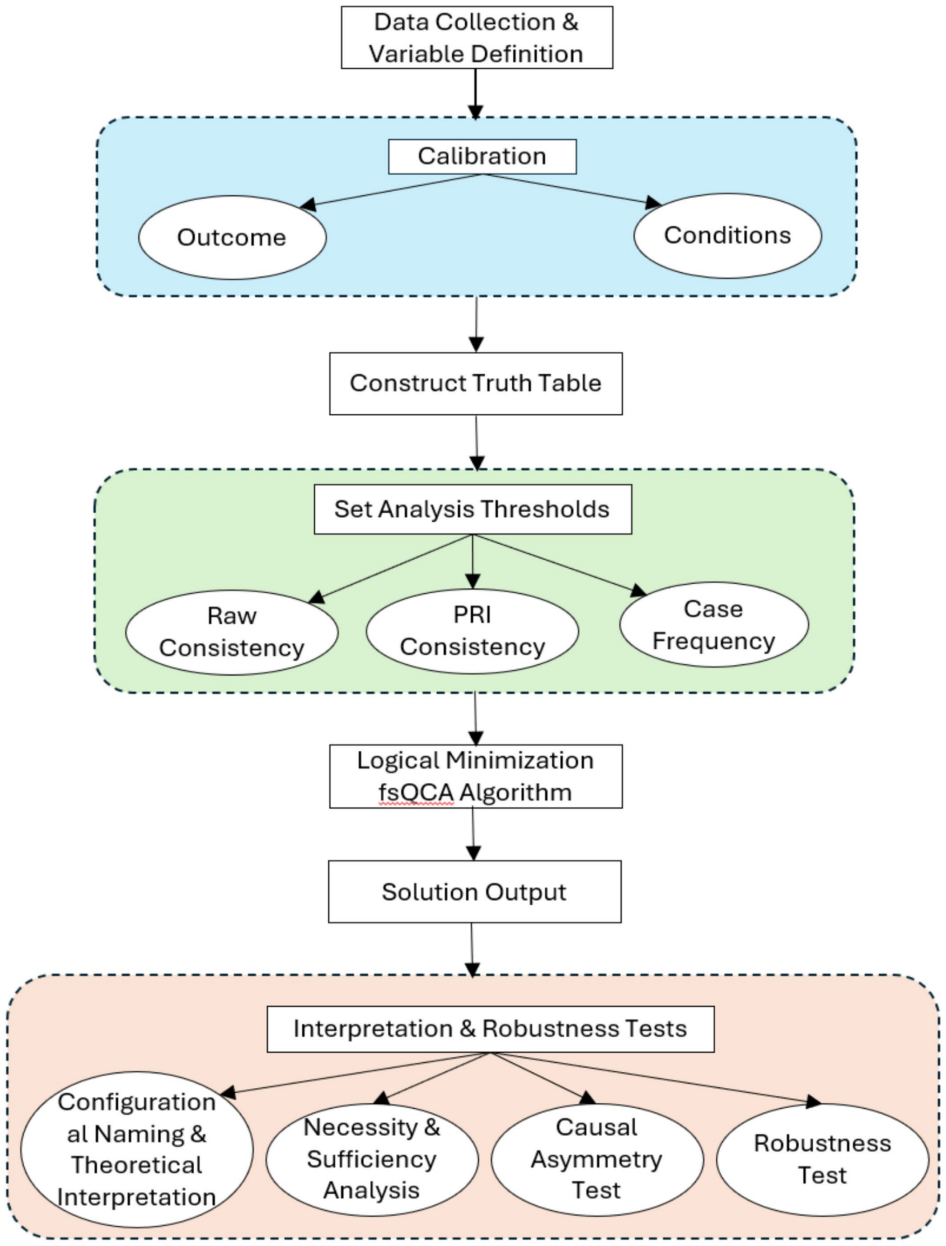
Flowchart of the fsQCA procedure.

Our fsQCA procedure followed the established protocols in the methodological literature (42, 43). The analytical process, summarized in Figure 2, involved four key steps: (1) calibration of conditions and outcome, (2) construction and analysis of the truth table, (3) logical minimization to derive solutions, and (4) interpretation of configurations and robustness checks. Each step is detailed below to ensure full transparency and replicability.

### 3.2 Data and calibration

This study utilizes data for the year 2022. This period was selected as it represents the most recent year for which a complete and consistent dataset for all variables across all 31 provincial-level regions is available from authoritative national statistical sources.

#### 3.2.1 Outcome variable

The dependent variable in this study is the PHC capacity at the provincial level, measured by the number of healthcare visits (outpatient and inpatient) per 10,000 population at primary healthcare institutions in 2022. This approach to measuring service delivery capacity is consistent with prior health services research (44). It provides a standardized means for cross-regional comparison. Data is sourced from the *China Health Statistical Yearbook 2023*. The total number of healthcare visits includes all patients seeking care at primary healthcare institutions, encompassing both outpatient and inpatient visits, thereby providing a comprehensive reflection of the service capacity of these institutions. An increase in the number of visits typically indicates a higher demand for primary healthcare services, reflecting both the health needs of the population and the level of trust residents place in PHC.

While we acknowledge that primary healthcare capacity encompasses multiple dimensions including quality, accessibility, and workforce, the core dependent variable in this study—the number of healthcare visits per 10,000 population—is justified as a key output indicator. From a systems theory perspective, it reflects the realized service volume of the PHC system, representing the equilibrium point between service supply (driven by resources and institutional arrangements) and population demand. According to resource dependence theory, the ability of PHC institutions to attract and serve a large patient population is a direct manifestation of their operational capacity and societal legitimacy, which is sustained by critical resource inputs. Therefore, in the context of investigating the configurational drivers of PHC system performance, this measure provides a valid, comparable, and outcome-focused metric across provinces.

#### 3.2.2 Condition variables

The level of economic development was measured by provincial per capita GDP in 2022, whereas population size was proxied by provincial population density in the same year. Educational attainment was captured by the average years of schooling per capita in 2022 (5). While not a direct measure of clinical competency, the average years of schooling serves as a key macro-level indicator of a region’s human capital foundation, which influences the supply, quality, and professional environment of the healthcare workforce. These three indicators, together with the urbanization rate, were obtained from the *China Statistical Yearbook 2023*. Healthcare expenditure was assessed using total healthcare expenditure in 2022, as reported in the *China Health Statistics Yearbook 2024*. Data on medical insurance fund revenue for 2022 were drawn from the *China Health Statistics Yearbook 2023*. Constrained by the availability of data, HDT was operationalized by the family doctor contract rate in 2022, compiled from official reports released by provincial health commissions. The family doctor contract rate is employed as it represents the core operational policy for implementing HDT in China, directly reflecting the system’s coverage and institutional roll-out.

#### 3.2.3 Calibration

Building on extant literature and supported by established theoretical and empirical insights, this study employs the direct calibration method to convert raw data into fuzzy set membership scores, fully integrating the data characteristics of each condition and the outcome (43, 45). Following the practice of Tao et al. the calibration anchors for the seven condition variables and the outcome variable (PHC capacity) were set as follows: the three thresholds—full membership, crossover, and full non-membership—were assigned at the 95th, 50th, and 25th percentiles of the sample data, respectively (46). The selection of the 95th (full membership), 50th (crossover point), and 25th (full non-membership) percentiles as calibration thresholds is grounded in both theoretical and empirical considerations. Theoretically, these percentiles allow us to clearly differentiate between cases that are unambiguously ‘in’ the set (top 5%), those that are ambivalent or typical (around the median), and those that are unambiguously ‘out’ of the set (bottom 25%). This aligns with the fsQCA convention of using the data distribution itself to inform set membership when external theoretical or substantive criteria are not strictly predefined (47). Empirically, these thresholds effectively capture the substantial variation across Chinese provinces without being overly sensitive to extreme outliers. Calibration of weak PHC capacity was achieved by taking the negation of the strong PHC capacity set. Detailed calibration anchors for all variables are presented in Table 1.

**Table 1:** Calibration of outcome and condition variables.

Outcome variable and theory	Conditions and outcomes	Calibration		
		Full membership	Crossover point	Full non-membership
Outcome Variable	PHC capacity	37141.50	10144.00	1359.00
Systems theory	Level of economic development	162360.00	70836.00	51711.00
	Urbanization rate	0.86	0.64	0.53
	Population size	1235	282	12
Resource dependence theory	Healthcare expenditure	6175.71	2081.85	426.17
	Medical insurance fund revenue	8377.24	3808.30	605.56
Institutional logic theory	Educational attainment	11.47	9.70	8.62
	HDT	0.99	0.80	0.72

## 4 Data analysis and empirical results

### 4.1 Necessary condition analysis

According to the QCA theoretical framework, a condition is deemed necessary if it is invariably present whenever the outcome occurs (43). It is crucial to distinguish between trivial and non-trivial necessary conditions, and our analysis adheres to this rigorous standard (42, 48). The results of the necessity analysis for both strong and weak PHC capacity are presented in Table 2. Findings reveal that, with the exception of high healthcare expenditure, all conditions exhibit consistency scores below the 0.90 benchmark. This suggests that high healthcare expenditure may potentially represent a necessary condition for strong PHC capacity. However, subsequent scatterplot analysis examining the relationship between the outcome variable and high healthcare expenditure indicates a dispersed distribution of data points, lacking clear clustering. Consequently, further empirical testing substantiates that high healthcare expenditure alone does not satisfy the criteria for a necessary condition in explaining the outcome variable.

**Table 2:** Necessary condition analysis.

Condition variables	Strong PHC capacity		Weak PHC capacity	
	Consistency	Coverage	Consistency	Coverage
High level of economic development	0.658	0.639	0.529	0.620
Low level of economic development	0.608	0.517	0.691	0.709
High urbanization rate	0.586	0.571	0.596	0.699
Low urbanization rate	0.691	0.586	0.634	0.649
Large population size	0.747	0.757	0.474	0.580
Small population size	0.586	0.481	0.802	0.793
High healthcare expenditure	0.921	0.826	0.481	0.520
Low healthcare expenditure	0.465	0.426	0.840	0.928
High medical insurance fund revenue	0.941	0.853	0.407	0.445
Low medical insurance fund revenue	0.388	0.351	0.866	0.947
High educational attainment	0.581	0.569	0.617	0.729
Low educational attainment	0.724	0.610	0.635	0.646
High degree of HDT	0.545	0.514	0.639	0.726
Low degree of HDT	0.710	0.620	0.572	0.603

### 4.2 Condition configurational analysis

It is important to distinguish between necessary conditions and core conditions within sufficient configurations, as they represent different causal relationships. A necessary condition must be present for the outcome to occur, acting as a prerequisite. In contrast, a core condition within a sufficient configuration is a pivotal element of a specific causal recipe that can produce the outcome; however, the outcome may also be achieved through other recipes that do not include this condition. This is a manifestation of equifinality. Thus, the empirical finding that high healthcare expenditure is not necessary but is core to several sufficient pathways is not a contradiction but rather a core insight from our configurational analysis: while provinces can achieve high PHC capacity without high expenditure (hence, it is not necessary), for those that follow the pathways we identified, it is very often a critical driving factor. The sufficiency analysis employs a truth table algorithm to identify all logically possible and empirically observed configurations (43). Considering the empirical distribution of cases within this study, thresholds were set as follows: Raw Consistency at 0.80, Proportional Reduction in Inconsistency (PRI) Consistency at 0.70, and Case Frequency at 1. A raw consistency threshold of 0.8 is the commonly accepted minimum for sufficiency analysis in QCA studies (43). The PRI consistency threshold of 0.7 and a case frequency of 1 were chosen to mitigate the risk of “simultaneous subset relations” (42). It is noteworthy that at these thresholds, no contradictory configurations (i.e., configurations associated with both high and low levels of the outcome) were identified, indicating a clear subset relationship for the configurations included in the solution. The truth table for strong PHC capacity is available in Appendix A.

This study conducts separate configurational analyses to elucidate the pathways leading to both strong and weak PHC capacity. In accordance with the theoretical imperatives of configurational methodology, the resultant configurations are subsequently labeled to facilitate interpretability and theoretical integration.

#### 4.2.1 Configurational pathways leading to strong PHC capacity

Table 3 delineates four configurational pathways that explicate strong PHC capacity. As indicated, the solution consistency stands at 0.917, demonstrating that 91.7% of cases conforming to these four configurations exhibit robust PHC capacity. The overall solution coverage is 0.716, reflecting that these configurations collectively account for 71.6% of the cases characterized by strong PHC capacity. Both the individual configuration consistencies and the aggregate solution consistency surpass the conventional threshold of 0.75, thereby affirming the reliability and validity of the empirical findings. Consequently, these four configurations are identified as sufficient condition sets for achieving strong PHC capacity. Notably, all four configurations underscore high healthcare expenditure as a core condition, highlighting the pivotal role of adequate financial investment in fostering the enhancement of PHC capacity. This study followed the heuristics of Furnari et al. to conceptualize and name the configurations as the Resource-Intensive Pathway, Fiscal-Led Pathway, and Institutional-Synergy Pathway (31).

**Table 3:** Configurational analysis of strong PHC capacity.

Condition configuration	Resource-intensive pathway		Fiscal-led pathway	Institutional-synergy pathway
	Configuration 1	Configuration 2	Configuration 3	Configuration 4
Level of economic development	●		⊗	●
Urbanization rate	●	⊗	⊗	
Population size	●	●		●
Healthcare expenditure	●	●	●	●
Medical insurance fund revenue	●	●	●	●
Educational attainment		⊗	⊗	●
HDT		●	⊗	⊗
Consistency	0.938	0.975	0.901	0.931
Raw coverage	0.498	0.362	0.435	0.433
Unique coverage	0.062	0.047	0.088	0.047
Solution consistency	0.917			
Solution coverage	0.716			

Following Fiss (11), Black circles indicate the presence of a condition, and circles with “×” indicate its absence. Large circles indicate core conditions; small ones, peripheral conditions. Blank spaces in dicate “do not care”.

##### 4.2.1.1 Resource-intensive pathway

Configuration 1 demonstrates a consistency of 0.938, a raw coverage of 0.498, and a unique coverage of 0.062, indicating that this configuration accounts for approximately 49.8% of cases exhibiting strong PHC capacity, with 6.2% of cases uniquely explained by this pathway. Configuration 2 demonstrates a consistency of 0.975, a raw coverage of 0.362, and a unique coverage of 0.047, indicating that this configuration accounts for approximately 36.2% of cases exhibiting strong PHC capacity, with 4.7% of cases uniquely explained by this pathway. In Configurations 1 and 2, population size, healthcare expenditure, and medical insurance fund revenue all constitute core conditions. In other words, the synergistic interaction among high healthcare expenditure, population size, and medical insurance fund revenue effectively enhances PHC capacity.

**Figure 3:**
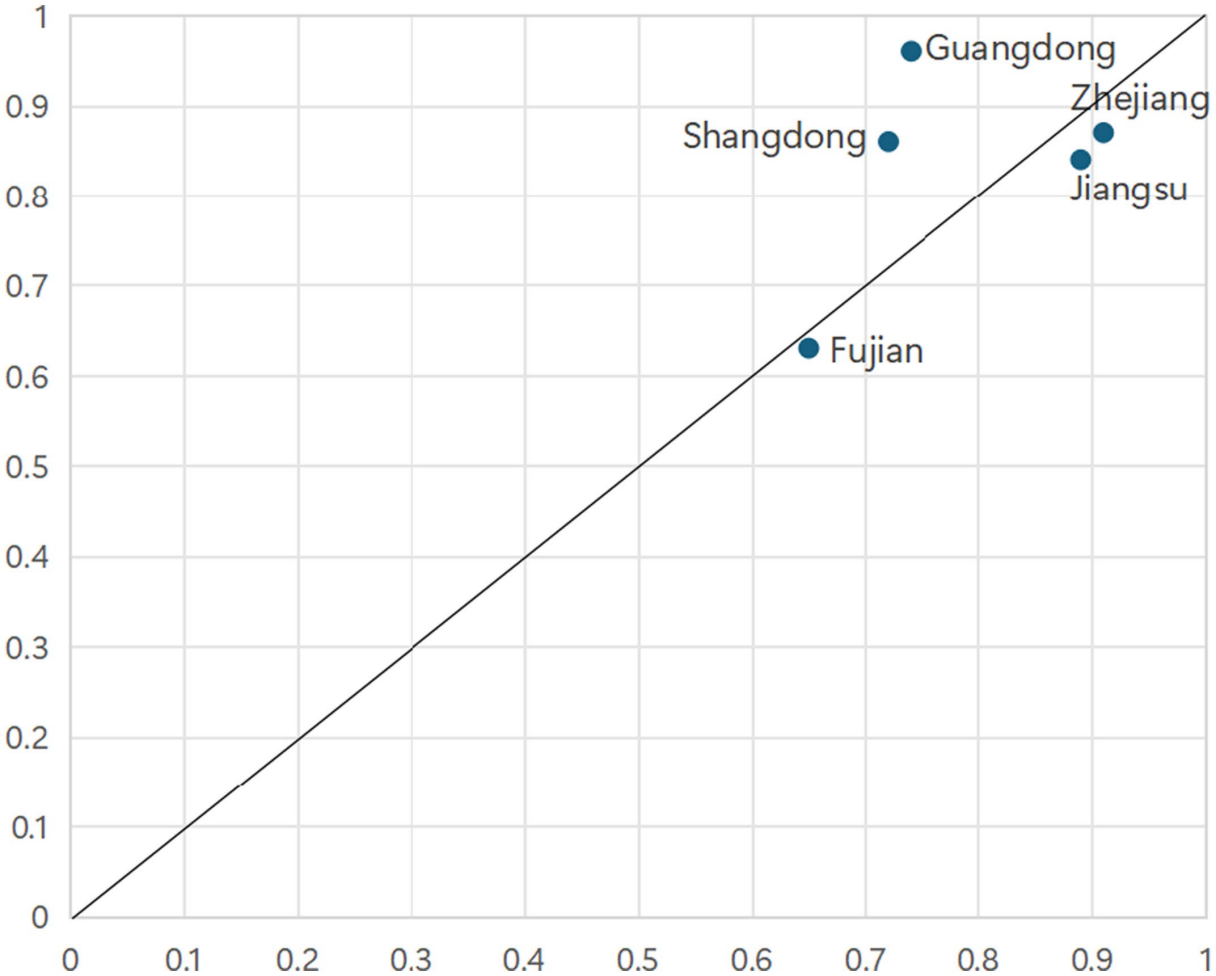
Cases explained by Configuration 1.

**Figure 4:**
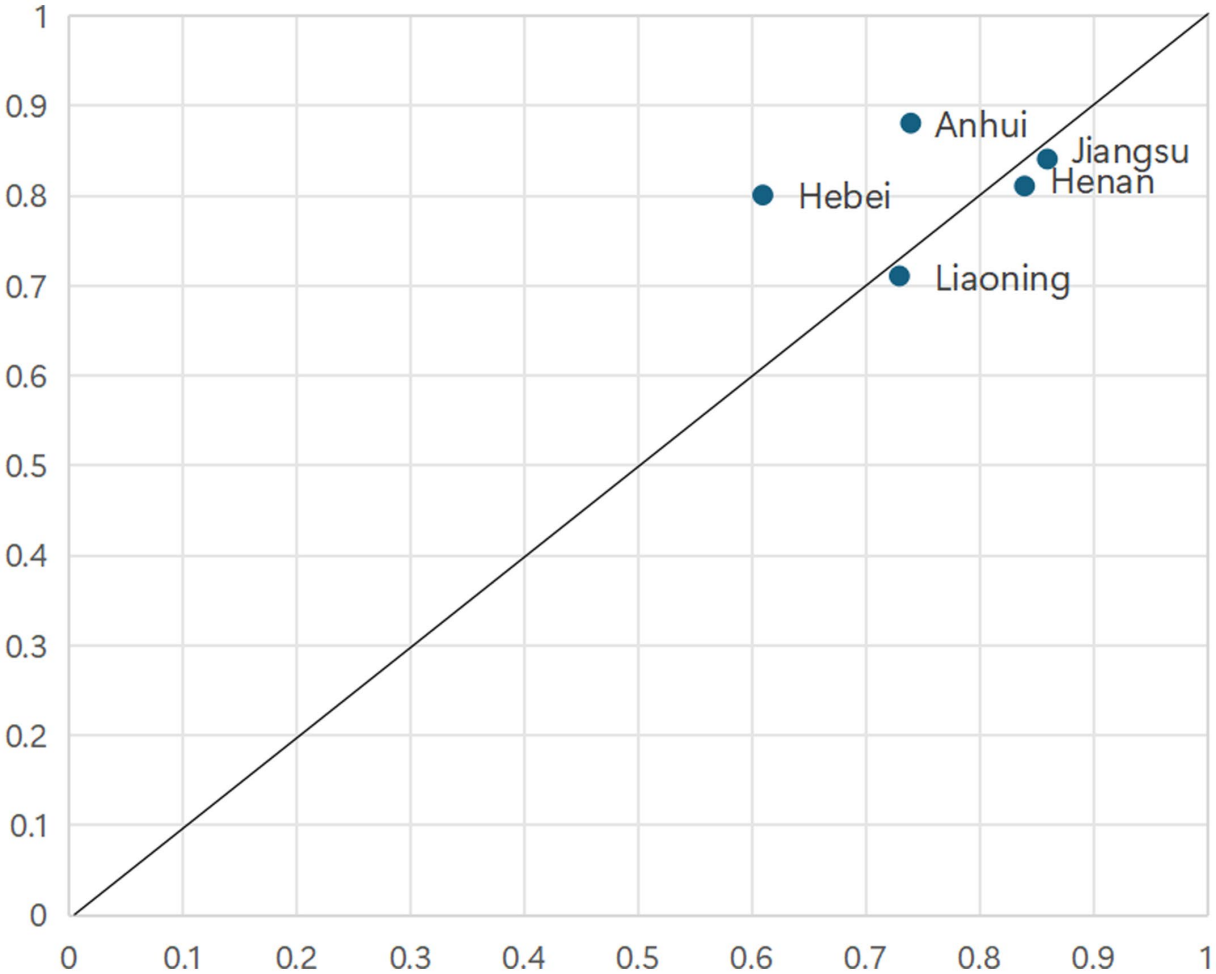
Cases explained by Configuration 2.

As illustrated in Figure 3, configuration 1 accounts for cases from eastern coastal provinces, including Guangdong, Jiangsu, Zhejiang, Shandong, and Fujian. These provinces are characterized by relatively developed economies, high urbanization rates, substantial medical insurance fund revenues, significant healthcare expenditures, and strong educational foundations. Taking Guangdong as an example, it ranks among the top regions nationally in terms of population density, possesses ample medical insurance fund revenue, and maintains considerable healthcare spending. Configuration 1 is exclusively adopted by affluent Eastern Coastal provinces. They leverage a “System-Resource Synergy,” where a superior macro-system (high economic development & urbanization) synergizes with abundant resource inputs. As shown in Figure 4, the cases explained by Configuration 2 comprise Henan, Hebei, Jiangsu, Liaoning and Anhui provinces. Configuration 2 represents a “Pure Resource-Driven” model, where massive resource inputs compensate for macro-system disadvantages. Taking Henan Province as an example, its population ranks third nationally. The province leverages this substantial population base to increase revenue for its medical insurance funds, thereby catalyzing an enhancement in primary healthcare capacity to meet the correspondingly high demand for medical services.

Configuration 1 and 2 together delineate a “Resource-Intensive Pathway” pathway. This pathway reveals a compelling compensatory mechanism within the integrated theoretical framework. The macro-contextual conditions outlined by Systems Theory—specifically, a less developed economy and, in some cases, lower urbanization or education—create a challenging environment for developing strong PHC capacity. However, this systemic disadvantage is effectively compensated for at the meso-resource level as per Resource Dependence Theory. A large population generates substantial service demand and potential risk-pooling for insurance, which, when coupled with robust financial inputs from both public coffers (Healthcare expenditure) and the social insurance system (Medical insurance fund revenue), creates a powerful resource-driven momentum. This configuration underscores that when the macro-context is less favorable, the sheer intensity of meso-level resource infusion—driven by demographic and fiscal forces—can overcome systemic barriers to achieve strong PHC capacity. The absence of HDT as a core condition in these paths further suggests that in such resource-intensive models, institutional fine-tuning (Institutional Logic Theory) may be secondary to the sheer volume of resource commitment.

##### 4.2.1.2 Fiscal-led pathway

The consistency score for Configuration 3 is 0.901, with a raw coverage of 0.435 and a unique coverage of 0.088, indicating that this pathway explains approximately 43.5% of cases characterized by strong PHC capacity, with 8.8% of cases uniquely attributable to this configuration. In Configuration 3, healthcare expenditure emerges as the sole core condition, while medical insurance fund revenue, economic development level, urbanization rate, and educational attainment exert comparatively limited influence on PHC capacity. HDT functions as an ancillary condition.

**Figure 5:**
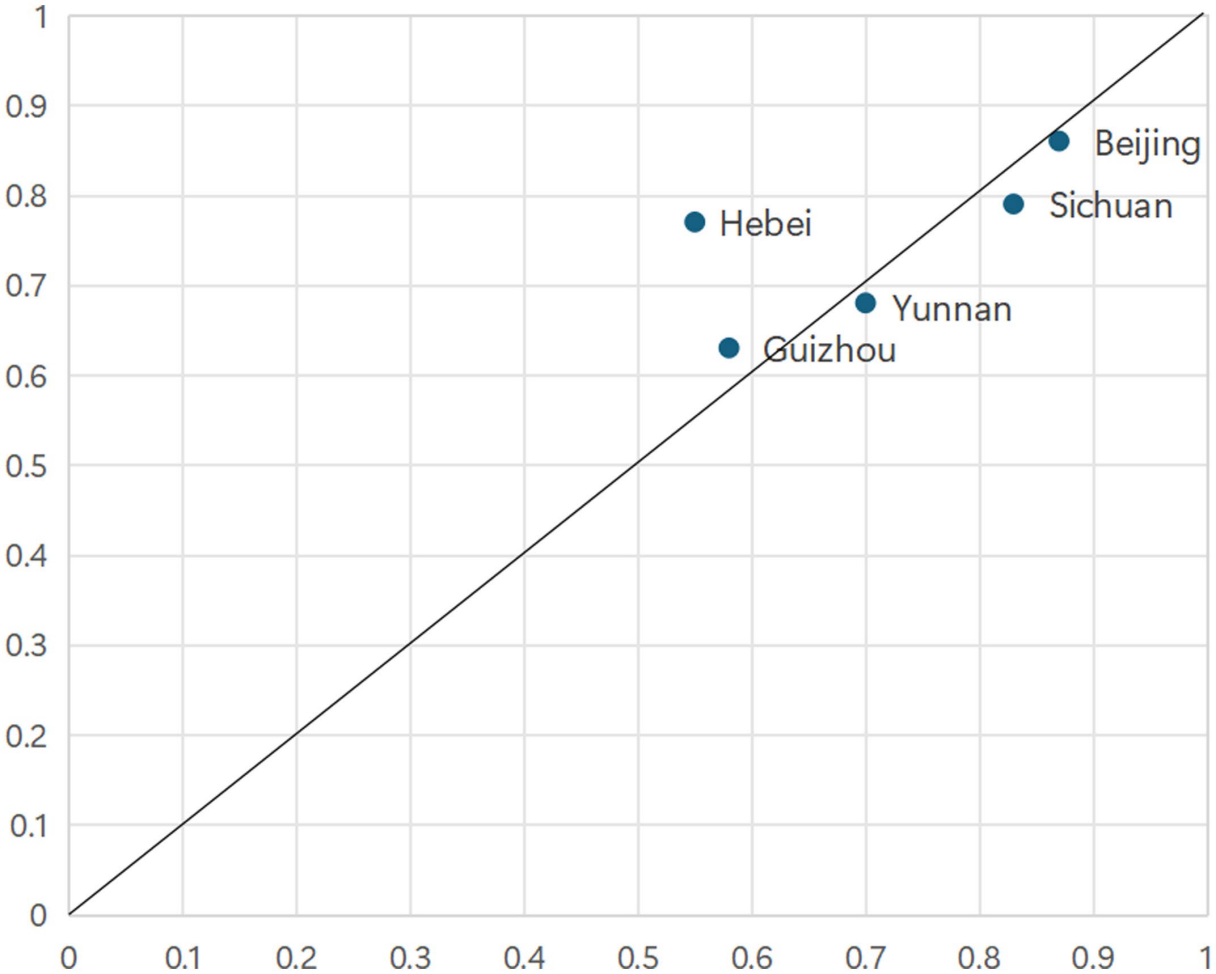
Cases explained by Configuration 3.

As presented in Figure 5, configuration 3 reveals that robust primary healthcare capacity is attainable under specific conditions, as exemplified by Hebei, Beijing, Sichuan, Guizhou and Yunnan provinces. These cases are characterized by a distinct set of conditions that collectively drive the enhancement of service capabilities. Configuration 3 is characteristic of Western and less developed provinces. It exemplifies a “Single-Core Fiscal Dependency” model, where direct central government fiscal transfers act as the sole, crucial pillar supporting PHC capacity in the absence of other strong conditions. Taking Hebei Province as a case in point, its provincial government released the “Implementation Plan for Further Improving the Healthcare Service System” in 2023. This plan underscores the role of social capital participation, guarantees government investment, and aims to enhance primary healthcare capacity.

Configuration 3 represents the “Fiscal-Led Pathway.” This pathway exemplifies a “single-core driving” pattern, the potency of which can be best understood through the lens of the multi-level framework. The macro-contextual conditions (Systems Theory) and the institutional logic of human capital (Educational attainment) are largely absent or peripheral. Similarly, the other key channel of resource dependence, medical insurance fund revenue is not a core driver. Despite these shortcomings across multiple theoretical dimensions, strong PHC capacity is still achieved. The key lies in the overwhelming dominance of a single, core meso-resource: high healthcare expenditure. This finding highlights that direct fiscal investment can, in certain contexts, substitute for weaknesses in the broader socioeconomic system and other resource channels. Although HDT is present as a peripheral condition—indicating a facilitative role for policy intervention—the primary engine remains unequivocally the financial input from healthcare expenditure. This demonstrates the paramount importance of the Resource Dependence channel within this specific configuration.

##### 4.2.1.3 Institutional-synergy pathway

Configuration 4 exhibits a consistency of 0.931, a raw coverage of 0.433, and a unique coverage of 0.047, indicating that it accounts for approximately 43.3% of cases exhibiting strong PHC capacity, with 4.7% uniquely explained by this configuration. In Configuration 4, population size, healthcare expenditure, and medical insurance fund revenue constitute the core conditions, supplemented by HDT as a peripheral condition. The HDT represents a pivotal strategy within China’s ongoing healthcare reform, aimed at optimizing medical resource allocation and delineating clear functional divisions among various levels of medical institutions. This approach seeks to strengthen PHC capacity and mitigate structural imbalances. Configuration 4 reveals that, despite low levels of economic development, urbanization, and educational attainment, provinces characterized by large populations and abundant resources—reflected in high healthcare expenditure and substantial medical insurance fund revenues—can achieve elevated PHC capacity through the active promotion of HDT.

**Figure 6:**
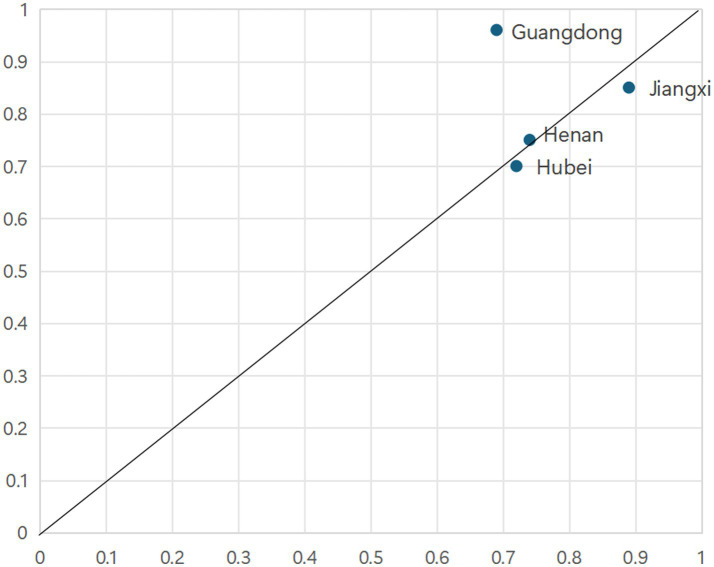
Cases explained by Configuration 4.

As illustrated in Figure 6, configuration 4 is sufficient in explaining the outcomes observed in Guangdong, Jiangxi, Henan and Hubei provinces. Configuration 4 represents an upgraded “Resource-Institution Synergy” model, where resource endowment is catalyzed by the institutional innovation of HDT to achieve efficiency gains. Exemplified by Hunan Province, regional efforts have spearheaded the implementation of the hierarchical diagnosis and treatment system and innovative models for chronic disease management, as documented in the 2021 Hunan Provincial Health Care Development Statistical Bulletin. These initiatives are marked by the establishment of 15 national-level integrated chronic disease prevention and control demonstration zones, 28 national mortality surveillance sites, and 122 tumor registry sites by the end of 2021.

Configuration 4 is labeled the “Institutional-Synergy Pathway.” This pathway offers a clear illustration of the synergistic interplay between the theoretical dimensions. Similar to the Resource-Intensive Pathway, it operates within a constrained macro-context (Level of economic development and Urbanization rate) but benefits from a favorable demographic structure and robust resource bases (Healthcare expenditure and Medical insurance fund revenue). What distinguishes this pathway is the pivotal role of institutional logic. The absence of a high degree of HDT implementation is a core condition here. This indicates that the institutional reform of HDT is not merely an add-on but a critical, active ingredient that interacts synergistically with resource endowments. In this pattern, the macro-structural constraints are overcome not just by resource abundance, but by a fundamental reconfiguration of the institutional logic governing healthcare delivery. The HDT policy effectively channels and optimizes the use of existing resources, demonstrating how a meso-resource advantage is amplified by a supportive micro-institutional mechanism to achieve the outcome, thereby fully embodying the spirit of the collaborative governance framework.

The “Institutional-Synergy Pathway” identified in this study offers a complementary perspective to the existing literature on China’s HDT reforms. For instance, Xiao et al. (39) highlighted significant challenges in establishing a graded diagnosis and treatment system in China, including low patient compliance and insufficient service capacity at the primary care level. Our findings, however, reveal that in provinces with large populations and substantial medical insurance fund revenues, the active implementation of HDT can serve as a critical institutional lever that effectively mobilizes existing resources and compensates for relative disadvantages in economic development or urbanization. This suggests that the success of HDT is not solely determined by the policy design itself, but is contingent on its synergistic combination with specific resource endowments. This configurational insight helps explain why HDT reforms yield significant benefits in some contexts but not others, thereby enriching the understanding of HDT’s conditional effectiveness (38, 39).

#### 4.2.2 Configurational pathways leading to weak PHC capacity

This study further examined the negation set of the outcome variable, specifically focusing on weak PHC capacity, to explore the phenomenon of causal asymmetry—a key tenet of set-theoretic methods where the paths to an outcome’s presence may differ from those to its absence (12, 43). The empirical analysis identified five distinct configurational patterns that contribute to weak PHC capacity. As detailed in Table 4, the solution consistency is 0.954, indicating that 95.4% of cases exhibiting these five configurations correspond to weak PHC capacity. Moreover, the overall solution coverage is 0.715, demonstrating that these five configurational pathways collectively account for 71.5% of the instances characterized by weak PHC capacity.

**Table 4:** Configurational Analysis of Weak PHC capacity.

Condition configuration	The “Systemic Resource Scarcity” pattern			The “Institutional-Resource Double Failure” pattern	
	Configuration 1	Configuration 2	Configuration 3	Configuration 4	Configuration 5
Level of economic development	⊗	⊗	●	⊗	●
Urbanization rate		⊗	●	●	●
Population size	⊗	●	⊗	●	●
Healthcare expenditure	⊗	⊗	⊗	⊗	
Medical insurance fund revenue	⊗	⊗	⊗	●	⊗
Educational attainment		●	●	●	●
HDT	●	⊗	⊗	⊗	●
Consistency	0.994	0.987	0.989	0.941	0.924
Raw coverage	0.459	0.217	0.271	0.224	0.278
Unique coverage	0.243	0.011	0.042	0.036	0.113
Solution consistency	0.954				
Solution coverage	0.715				

Following Fiss (11), Black circles indicate the presence of a condition, and circles with “×” indicate its absence. Large circles indicate core conditions; small ones, peripheral conditions. Blank spaces in dicate “do not care”.

Our in-depth analysis of the pathways to weak PHC capacity reveals two predominant, theoretically distinct failure modes that clearly demonstrate causal asymmetry (12). These are not simply the inverse of the success pathways but represent unique recipes for failure.

##### 4.2.2.1 The “Systemic Resource Scarcity” pattern

Configuration 1 reveals that under conditions of low economic development, limited population size, insufficient healthcare expenditure, and reduced medical insurance fund revenue, the presence of HDT alone is insufficient to foster strong PHC capacity. Configuration 2 demonstrates that even with a large population and high educational attainment, low levels of economic development, urbanization, healthcare expenditure, medical insurance fund revenue, and HDT collectively hinder the enhancement of PHC capacity. Configuration 3 suggests that low economic development, population size, HDT, healthcare expenditure, and medical insurance fund revenue, despite concurrent high urbanization and educational levels, fail to generate strong PHC capacity. The “Systemic Resource Scarcity” Pattern (evident in configurations 1, 2, and 3 from Table 4) is defined by the core absence of both healthcare expenditure and medical insurance fund revenue. This demonstrates that the dual deprivation of fiscal and social insurance resources constitutes a sufficient condition for failure on its own terms, regardless of other contextual factors. This is asymmetrical to the success paths, where the presence of high expenditure is core, but its absence is part of a unique, failure-inducing recipe.

##### 4.2.2.2 The “Institutional-Resource Double Failure” pattern

Configuration 4 highlights that in contexts marked by inadequate healthcare expenditure, weak economic development, and insufficient HDT, favorable urbanization rates, population sizes, and educational attainment do not translate into enhanced PHC capacity. Configuration 5 indicates that when economic development, urbanization rate, population size, HDT, and educational attainment are all low, the absence of healthcare expenditure precludes the development of robust PHC capacity. The “Institutional-Resource Double Failure” Pattern (evident in configurations 4 and 5) reveals that the combination of low healthcare expenditure (resource failure) and the absence of HDT implementation (institutional failure) creates a distinct pathway to weak capacity. Crucially, this specific combination of institutional and resource deficiencies does not appear in any of the strong-capacity configurations, providing clear evidence of causal asymmetry. It shows that failure has its own logic, where the lack of a key institutional reform (HDT) synergizes with resource scarcity to produce a poor outcome.

All in all, the failure pathways reveal that the absence of key drivers is not always symmetrical to their presence. The most striking finding is the overwhelming and consistent role of insufficient healthcare expenditure across failure configurations. While high expenditure was a core element in all successful paths, its absence emerges as an almost universal culprit in failure, appearing as a core condition in four out of five pathways. This asymmetry highlights that avoiding failure may have a lower but more non-negotiable barrier: ensuring a minimum threshold of financial investment. In contrast, achieving success allows for more flexibility and substitutability among conditions. This nuanced understanding enriches our multidimensional collaborative governance framework by illustrating that the mechanisms of system failure are distinct from those of system success, thereby offering more precise and differentiated policy guidance.

## 5 Robustness test

This study undertook a series of robustness checks on the antecedent configurations associated with strong PHC capacity (48).

Firstly, the original dependent variable was replaced with a pure supply-side indicator: “the number of health technicians per 10,000 population in primary healthcare institutions” (data sourced from the China Health Statistical Yearbook 2023). This measure directly reflects the human resource capacity of the PHC system, independent of patient visitation patterns. The new variable was calibrated using the same direct method, with anchors set at the 95th, 50th, and 25th percentiles. The solution consistency for achieving high PHC personnel density was 0.906, and the solution coverage was 0.694. The results strongly corroborate our original findings. No single condition was necessary for high PHC personnel density. High Healthcare Expenditure re-emerged as a core condition in the majority of the sufficient configurations leading to high PHC personnel density. The overarching logic of the configurational pathways remained broadly consistent, reinforcing the central role of financial investment and its interplay with other conditions. This demonstrates that the central role of sustained financial investment is a robust driver of PHC capacity, whether capacity is measured as a system output (visits) or a key system input (personnel).

Secondly, we conducted a sensitivity analysis by recalibrating our data using the 75th, 50th, and 25th percentiles as alternative anchors. The results of this analysis confirm the robustness of our primary findings. While the raw and unique coverage values saw minor fluctuations, the core conditions in each pathway remained identical, and the overall solution consistency remained high (0.910). This demonstrates that our conclusions are not dependent on the specific choice of the 95th/25th percentiles and are methodologically robust.

Thirdly, elevating the consistency threshold from 0.80 to 0.85 did not materially alter the resultant configurations, which continued to demonstrate stability. Thirdly, increasing the case frequency threshold from 1 to 2 produced configurations that remained substantively consistent with the original findings.

Finally, to address potential heterogeneity in resource endowments between the four directly administered municipalities—Beijing, Shanghai, Tianjin, and Chongqing—and other cities, these municipalities were excluded from the sample. The subsequent analysis yielded configurations congruent with those previously identified. Collectively, these robustness assessments substantiate the reliability and validity of the study’s findings.

## 6 Conclusion and future perspectives

### 6.1 Conclusion

Amid the ongoing advancement of national healthcare reform, PHC capacity has taken on a critical role in safeguarding population health, improving public health literacy, and fostering social cohesion. Guided by a multidimensional framework integrating systems, resource dependence, and institutional logic theories, this study employs fsQCA on data from 31 Chinese provinces. This investigation assesses the influence of seven key conditions—economic development level (resource supply foundation), urbanization rate (socio-structural characteristic), population size (service demand pressure), healthcare expenditure (fiscal guarantee capacity), medical insurance fund revenue (payment system support), educational attainment (human capital reserve), and hierarchical diagnosis and treatment (HDT) system implementation (institutional innovation efficacy)—on PHC capacity, with the aim of elucidating the configurational pathways and underlying mechanisms that drive capacity enhancement. By applying fsQCA to the domain of health policy, this study extends the application of the qualitative comparative analysis method and demonstrates the value of a neo-configurational perspective for tackling complex social phenomena in public health, offering a viable alternative to conventional correlational methods (11).

Empirical findings indicate that: first, none of the seven examined conditions individually constitutes a necessary prerequisite for strong PHC capacity. Second, elevated healthcare expenditure emerges as a pivotal core condition across four distinct configurational pathways. Third, three distinct configurational archetypes driving the strengthening of PHC capacity are identified: the “Resource-Intensive Pathway” pathway, indicative of resource compensation dynamics; the “Fiscal-Led Pathway” pathway, characterized by a single-core driving force; and the “Institutional-Synergy Pathway” pathway, exemplifying the synergistic interaction between resource availability and institutional innovation in the context of economic constraints. This study enriches the understanding of the multifaceted pathways and mechanisms underpinning capacity development, thereby advancing the strategic construction of primary healthcare systems and enhancing the quality and efficiency of services at the grassroots level. Finally, the pathways leading to weak PHC capacity are not mere inverses of the successful ones. We identified two independent failure modes—“Systemic Resource Scarcity,” characterized by the fatal dual absence of healthcare expenditure and medical insurance funds, and “Institutional-Resource Double Failure,” where the lack of fiscal investment coincides with failed HDT reforms. This demonstrates that the mechanisms preventing failure are distinct from those driving success.

### 6.2 Practical implications

Against the backdrop of regional developmental disparities in China, the findings of this study offer the following three practical implications for enhancing PHC capacity.

First, it is crucial to strengthen fiscal support as a central element, facilitated by a multi-stakeholder “Healthcare Expenditure+” coordination mechanism. Since healthcare expenditure is a core yet interdependent condition, provincial governments should prioritize stable fiscal investments while fostering synergistic “Healthcare Expenditure+” mechanisms. However, as healthcare expenditure alone is insufficient, policy frameworks must simultaneously implement complementary measures. These include enhancing payment capacity through elevated pooling levels of medical insurance funds and improving public health literacy through educational development. Such an integrated approach can create a “multiplier effect” between fiscal support and other contributing conditions.

Second, it is essential to tailor pathway selection to local conditions and implement regionally differentiated strategies. Policymakers should ground their strategies in empirical realities to select pathway combinations that exhibit the highest contextual fit. For the “Resource-Intensive Pathway” (e.g., eastern provinces), the focus is on “System Optimization.” Policies should promote “Internet + Healthcare” initiatives to improve efficiency and refine DRG/DIP payment reforms in urban medical consortia. These measures will further optimize resource allocation within these already robust systems. For the “Fiscal-Led Pathway” (e.g., underdeveloped areas reliant on fiscal transfers), the focus is on “Targeted Capacity Building.” This requires increasing targeted central transfers for PHC infrastructure and staffing, alongside expanding critical illness insurance coverage. Together, these actions reduce out-of-pocket expenditures and prevent poverty due to illness. For the “Institutional-Synergy Pathway” (e.g., central provinces actively implementing HDT reforms), the focus is on “Institutional Leverage.” We recommend “Implementing bundled payments for chronic disease management” under the HDT framework, which financially incentivizes primary care for managing these conditions. Additionally, we suggest “strengthening competency-based training for family doctors” to enhance the quality of the HDT “gatekeeper.”

Third, our findings provide concrete mechanisms for advancing the Healthy China 2030 goals. The principle of equifinality—that multiple pathways can achieve strong PHC—directly supports Healthy China 2030’s goal of equitable, accessible, and lifelong healthcare for all. The identified pathways offer a menu of options for different regions to achieve this common goal. The study also resonates with the WHO’s IPCHS framework by demonstrating its contextual implementation. The IPCHS framework calls for coordinated, multi-level services tailored to population needs. Our configurational analysis demonstrates that in China, this “people-centered” outcome can be achieved through different recipes—whether through strong fiscal channels, institutional restructuring like HDT, or their combination. This underscores that there is no one-size-fits-all approach to building integrated care. Instead, low- and middle-income countries possess a toolkit of key conditions: financial investment, insurance systems, and institutional reforms. The key is to adaptively combine these elements based on local contexts, ultimately building strong, people-centered primary care.

In conclusion, the enhancement of PHC capacity requires the establishment of a flexible governance framework, characterized by the dual pillars of ensuring core condition guarantees and selecting contextually adaptive pathways. By fortifying foundational capacities through robust fiscal support and implementing precisely targeted, region-specific strategies, a systemic transformation and balanced advancement of healthcare service capabilities can ultimately be realized.

### 6.3 Research limitations and future directions

This study acknowledges several limitations. To begin with, due to constraints in data availability, this study relies on cross-sectional data from 2022, which, while suitable for identifying configurational pathways at a specific point in time, limits our ability to observe the temporal stability and dynamic evolution of these pathways. The causal inferences we draw are about the relationships observed in this period. Future research would greatly benefit from employing a longitudinal fsQCA (or a panel QCA) approach with multi-year data. Such a design could explore how configurational pathways shift over time in response to policy changes and socioeconomic development, thereby providing deeper insights into the long-term mechanisms of PHC capacity enhancement that this reviewer rightly highlighted. Second, the utilization of provincial-level administrative units as the analytical cases potentially masks substantial intra-provincial heterogeneity, including urban–rural disparities and geographic segmentation. Third, although supplemented by a robustness check, our primary measure of PHC capacity leans towards realized service utilization. Future research would benefit from constructing a comprehensive index that integrates multiple dimensions, such as personnel density, facility infrastructure, and service quality metrics, to provide a more holistic measurement of PHC capacity. Finally, this study employed the family doctor contract rate as a proxy to measure HDT due to data availability and its alignment with core national policy. While this indicator effectively reflects the system’s coverage and institutional rollout, it may not fully capture the qualitative dimensions of HDT implementation, such as the actual content and quality of services provided under the contracts, patient adherence to the referral system, or the clinical competence of family doctors. Future research would benefit from incorporating multi-dimensional indicators—such as the rate of two-way referrals between primary institutions and hospitals, or patient satisfaction with contracted services—to more comprehensively assess the implementation quality and effectiveness of the HDT system.

## Data Availability

The raw data supporting the conclusions of this article will be made available by the authors, without undue reservation.

## Appendix

Appendix A

Truth tables for strong PHC capacity.

**Table:**

X1	X2	X3	X4	X5	X6	X7	Y	Raw consistency
1	1	1	1	1	1	1	1	0.983
1	0	1	1	1	0	1	1	0.980
1	1	1	1	1	0	1	1	0.980
0	0	1	1	1	0	1	1	0.973
0	0	1	1	1	0	0	1	0.954
1	0	1	1	1	1	0	1	0.949
1	1	1	1	1	0	0	1	0.941
1	1	1	1	1	1	0	1	0.927
0	0	0	1	1	0	0	1	0.911
0	0	0	1	1	0	1	1	0.898
1	0	0	0	1	0	0	1	0.884
0	0	0	0	1	0	0	1	0.859
0	1	1	0	1	1	0	1	0.857
1	1	1	1	0	1	1	0	0.782
1	1	1	0	0	1	1	0	0.773
0	0	1	0	0	1	0	0	0.771
1	1	0	0	0	1	0	0	0.623
0	1	0	0	0	1	1	0	0.602
0	0	0	0	0	1	1	0	0.581
0	1	0	0	0	0	1	0	0.559
0	0	0	0	0	0	1	0	0.426

X1, Level of economic development; X2, Urbanization rate; X3, Population size; X4, Healthcare expenditure; X5, Medical insurance fund revenue; X6, Educational attainment; X7, HDT; Y, Strong PHC capacity. No contradictory configurations (where the same configuration is associated with both the presence and absence of the outcome) were encountered.
